# Implication of the Extracellular Matrix in Metastatic Tumor Cell Dormancy

**DOI:** 10.3390/cancers16234076

**Published:** 2024-12-05

**Authors:** Chloe Redoute-Timonnier, Patrick Auguste

**Affiliations:** University of Bordeaux, INSERM, BRIC, U1312, MIRCADE Team, F-33000 Bordeaux, France

**Keywords:** tumor cell dormancy, tumor cell reactivation, extracellular matrix, metastatic niche, dormant tumor cell therapy

## Abstract

Metastasis is the main cause of cancer-related deaths. The formation and growth of metastasis is a multistep process. Tumor cells extravasating in the secondary organ are in contact with a new microenvironment and a new extracellular matrix (ECM), called the metastatic niche. Some components of the ECM, such as periostin, can induce tumor cell growth in macrometastasis. In contrast, other components, such as Thrombospondin 1 (TSP-1), can maintain isolated cells in a dormant state. During dormancy, intracellular signaling activation, such as p38, maintains tumor cells arrested in the cell-cycle G0 phase for years. At any moment, stress can induce ECM modifications and binding to their specific receptors (mainly integrins) and reactivate dormant tumor cell growth in macrometastasis. In this review, we describe the tumor microenvironment of the different niches implicated in tumor cell dormancy. The role of ECM components and their associated receptors and intracellular signaling in the reactivation of dormant tumor cells in macrometastasis will be emphasized. We also present the different methodologies and experimental approaches used to study tumor cell dormancy. Finally, we discuss the current and future treatment strategies to avoid late metastasis relapse in patients.

## Simple Summary

Metastasis, the growth of secondary tumors at distant sites, is responsible for the majority of cancer-related deaths. Most of the metastasis growth appears at the same time as the primary tumor or some years after treatment. Nevertheless, very late metastasis can develop, sometimes more than a decade after the end of treatment. At the origin of this late metastasis are isolated dormant tumor cells in the targeted secondary organ. The extracellular matrix of the metastasis-targeted organ plays an important role in maintaining tumor cells in dormancy or in reactivating tumor cell growth.

## 1. Introduction

Despite increasing advances and knowledge about its appearance and the mechanisms behind its formation, metastasis is the cause of most cancer-related deaths. Tumor cell dissemination occurs much earlier than previously estimated along the tumor development, and in rare cases, metastatic implantation can be detected before the primary tumor [1,2,3]. The multistep molecular low efficient cascades giving birth to metastasis formation are now well established [4]. Cancer cells in the primary tumor invade the adjacent normal tissue, intravasate into lymphatics and/or blood vessels, and develop strategies to survive in these hostile fluidic microenvironments. Then, they can extravasate from the vessel to enter the targeted secondary organ (soil in the “seed and soil” theory, see below) and primarily develop small colonies referred to as micrometastasis [4]. In the colonized organs, cancer cells called disseminated tumor cells (DTCs) are in a new hostile environment and surrounded by unconventional normal cells and extracellular matrix (ECM) compared to their tissue of origin. In targeted organs, DTCs can either develop macrometastasis or stay in a “non-proliferating” dormant state as isolated cells or as micrometastasis. The concept of “tumor dormancy” was first reported at the beginning of the twentieth century [5], although in 1954, Hadfield was the first to use the name “dormant cancer cell” for cells in temporary mitotic arrest [6]. More than 70 years later, two main dormant states have been described [7]. The first, referred to as “tumor mass dormancy” or “tumor dormancy”, implicates an equilibrium between proliferating and dying tumor cells in small clusters or micrometastases, resulting in the survival of a small population of cells with no tumor mass increase. This is caused by the incapacity of tumor cells to recruit new blood vessels, also known as neo-angiogenesis, or to the action of immuno-repressive mechanisms [8,9]. The second, called “tumor cell dormancy”, implicates non-proliferating cells, which stay quiescent in the cell-cycle G0 phase [7]. However, in contrast with senescent cells, which are irreversibly arrested in the G0 phase, dormant cells can, in certain conditions, be reactivated and re-enter the cell cycle [7]. Since it was reported that leukemic senescent cells can re-enter the proliferation program [10], the question about the limit between cell dormancy and senescence remains open. Importantly, many anticancer compounds used in clinics can induce tumor cell dormancy and drug tolerance or resistance [11]. These dormant cells are suspected to be at the origin of late recurrence, many years or decades after the seeming cure following treatment of the primary tumor. In breast cancer, half of the metastatic disease appears after 5 years of tumor-free survival [12] and 13–41% of the patients develop metastasis more than 5 years after the primary diagnosis [13,14]. Dormant cells have been found in most solid cancers, such as breast, prostate, lung, colon, and kidney carcinomas, but also in melanoma and in hematological cancers such as leukemia and myeloma [7]. The notion of tumor cell dormancy was further supported by organ transplantation-related pathologies. At the beginning of organ transplantations, donors with a cancer history were not accepted as organ donors. Due to the penury of organs, patients with a cancer history were finally accepted if they had a long disease-free period. Unfortunately, several patients who received an organ from these donors developed the same cancer as the donor [15]. Unexpectedly, donors with a history of glioblastoma, without a shunt from the brain to the abdomen and supposedly without metastasis outside the brain, were also able to transmit glioblastoma to patients transplanted with numerous peripheral organs, suggesting the presence of glioblastoma dormant cells outside the brain [15].

Tumor cells have been considered for a long time as the most critical component of tumor development. We now know that the tumor microenvironment (TME) is at least as important as tumor cells. The TME is composed of different types of cells (endothelial cells, immune cells, fibroblasts…) and non-cellular components, mostly constituting the ECM. The ECM is composed of proteins (collagens, elastin…), glycoproteins (laminins, fibronectin…), proteoglycans (glypican, syndecan, perlecan…), and glycosaminoglycans (hyaluronic acid) [4,16]. Fibroblasts or fibroblast-derived cells are the main cellular providers of ECM components; however, cancer cells and other cells from the TME can also produce and secrete ECM molecules. The ECM is organized into a basement membrane (BM) and interstitial matrix. The BM, which is essentially composed of laminins, type IV collagen, nidogen (entactin), and heparan sulfate proteoglycans, is a thin protein matrix supporting epithelial cell adhesion and cell-matrix signaling. The interstitial matrix forms a 3D complex network of different interconnected molecules that are constantly reshaping. This ECM is directly in contact with tumor cells and provides a protein basement for tumor cells’ anchorage. ECM components bind cell surface receptors such as integrins and participate in intracellular signaling inducing tumor cell division, migration, survival, and/or differentiation. Another important function of the ECM is to serve as a reservoir for regulatory factors such as Vascular Growth Factors (VEGFs), Fibroblast Growth Factors (FGFs), Transforming Growth Factors (TGFs), Epidermal Growth Factor (EGF), Platelet-Derived Growth Factors (PDGFs), and others, which are all involved in tumor development [17]. The composition of the ECM is continuously influenced by the synthesis, modification, and degradation of its components [4]. The Lysyl Oxidase (LOX) family proteins are secreted enzymes implicated in collagens and elastin modifications allowing cross-linking of the molecules [18]. On the other hand, different extracellular enzymes are able to digest ECM molecules, such as heparinases, cathepsins, Matrix Metalloproteinases (MMPs), and A Disintegrin And Metalloproteinase with Thrombospondin Motifs (ADAMTSs) [16]. MMPs are a group of enzymes that break down different components of the ECM, such as collagens, elastin, and proteoglycans. They are essential for tissue remodeling, wound healing, and development. MMPs contribute to both normal physiological processes and pathological conditions, including cancer metastasis [19]. Moreover, digestion of the ECM by a protease releases growth factors implicated in tumor development [17,19].

In the “seed and soil” theory of metastasis, primary tumor cells and TME induce a profound remodeling of the ECM of the targeted organ to define a permissive or restrictive microenvironment for DTCs called the pre-metastatic niche [20]. Depending on the microenvironment of the niche (soil), circulating DTCs intravasate (seed) and if the microenvironment is favorable, they can proliferate and develop macrometastasis. If not, they can stay quiescent in a dormant state. Later, when the remodeling of the niche leads to a permissive microenvironment, dormant tumor cells reactivate, proliferate, and develop macrometastasis [20,21].

Recently, Phan and Croucher have postulated six different hallmarks for cancer cell dormancy [7]: (1) the future dormant tumor cells first occupy a niche and develop interactions with the niche microenvironment, (2) in the niche, the tumor cells are arrested in G0 phase and undergo a cellular reprogramming, (3) the dormant tumor cells are resistant to drugs active in proliferating cells, (4) the dormant tumor cells activate an immune evasion mechanism, called immune cloaking, to be hidden from the immune system and to induce long-term dormancy, (5) dormant tumor cells can be reactivated to proliferate giving a metastasis relapse, and (6) in the niche, the metastatic tumor cells can again return in a dormant state.

In this review, we describe the metastatic niches and the different methodologies to study the metastatic niche modulators implicated in tumor cell dormancy or reactivation. Then, we show the importance of ECM molecules specifically expressed in the metastatic niche and implicated in tumor cell dormancy maintenance or those triggering reactivation and metastasis growth. We also highlight the role of different ECM receptors present on the tumor cell surface and implicated in the above mechanisms. Then, we discuss the different treatment strategies proposed to avoid late recurrence of tumor metastasis.

## 2. Metastatic Niches in Different Organs

The metastatic niche was first characterized as an area enhancing the efficiency of metastasis in the targeted secondary organ [22]. Later, Ghajar and colleagues characterized a perivascular metastatic niche (perivascular niche, PVN) where tumor cells might survive in a dormant state [23]. Then, in the bone marrow, the hypoxic hematopoietic stem cell niche, rich in osteoblasts and called the endosteal niche, was shown to play the same role [24].

**Figure 1:**
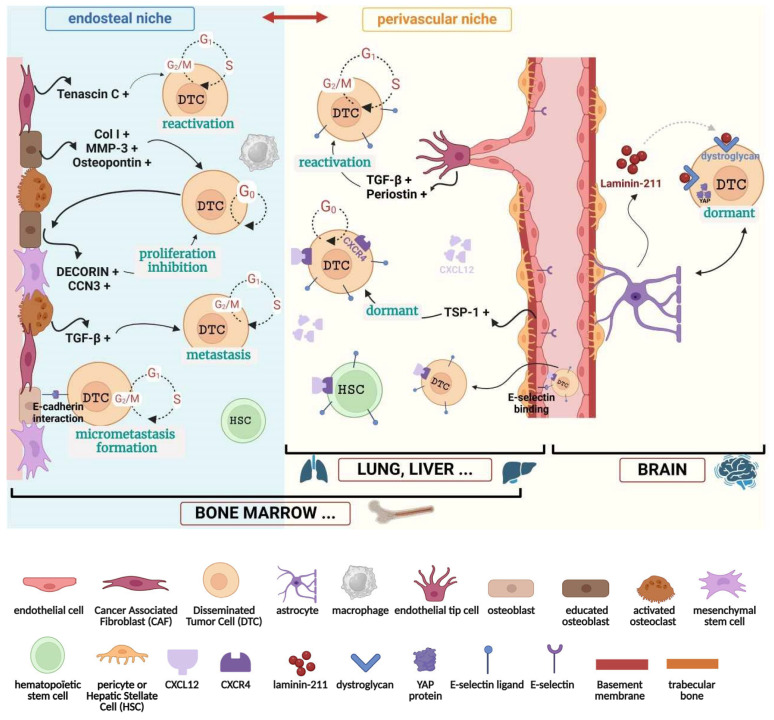
Cell composition of the endosteal and perivascular niches. The perivascular and endosteal niches are found in bone marrow, while the perivascular niche seems to be specific for the lung, liver, and brain. However, a large heterogeneity exists between endothelial cells from different organs [47]. Some factors involved in tumor cell dormancy or reactivation are presented. Figure created in BioRender. Redouté-Timonnier, C. (2024) https://BioRender.com/q13u172 (accessed on 1 December 2024).

The PVN was first characterized by Ghajar and colleagues in 2013 [23]. This niche is composed of cellular and non-cellular components. Endothelial cells forming the vessel are surrounded by mural cells, pericytes in capillaries, and smooth muscle cells in larger vessels (Figure 1). Endothelial and mural cells are separated by a BM. Depending on the organ, the composition of BM may differ. In the lungs, the endothelial BM is essentially composed of collagen type IV and XVIII, laminins, perlecan, nidogen, agrin, and versican and can sometimes contain Thrombospondin I (TSP-1) molecules. Pericytes and/or smooth muscle cells can secrete more ECM components such as collagen I and III, tenascin C, or fibronectin [25]. Most of these ECM molecules have a role in tumor cell dormancy or tumor cell reactivation (see below). Dormant breast tumor DTCs reside near the microvasculature of the lungs, bone marrow, or brain. Using an organotypic model of PVN, TSP-1 was shown to mediate tumor cell dormancy. TSP-1 is secreted by endothelial cells of the stable vasculature. In contrast, during angiogenesis, the tip cell secretes Transforming Growth Factor Beta 1 (TGF-β1) and periostin, allowing reactivation of the dormant tumor cells, their proliferation, and metastatic development (Figure 1) [23]. The homing of breast tumor cells to the bone marrow PVN is mediated by endothelial cell E-selectin, which binds E-selectin ligands expressed at the surface of tumor cells. The mobilization of dormant tumor cells from the PVN to the peripheral blood is mediated by C-X-C motif chemokine Receptor 4 (CXCR4) inhibition, suggesting that the axis CXCR4/CXCL12 (CXC motif chemokine 12) is crucial to anchor dormant tumor cells in the PVN (Figure 1). The same mechanisms of homing/mobilization have been observed with Hematopoïetic Stem Cells (HSC) [26]. Whether or not these homing/mobilization mechanisms are used for cancer cell dormancy in the PVN of other organs is unknown. In bone marrow PVN, breast cancer cells are protected from apoptosis induced by chemotherapy (e.g., doxorubicin plus cyclophosphamide, paclitaxel) in a cell quiescence-independent manner since tumor cells are equally killed by the drugs whether they express p27 or not. These data suggest that components of the PVN protect tumor cells from chemotherapy independently of the quiescent state of tumor cells [27]. In the brain, PVN is associated with interactions between the end feet of astrocytes and dormant tumor cells. The brain PVN is composed of endothelial cells in contact with a BM composed of collagen IV, laminin-411 and -511, nidogen, and perlecan. Between pericytes and the astrocytic feet, a BM enriched in laminin-211 can be found (Figure 1) [28]. In the presence of DTC, laminin-211 binds to dystroglycan at the tumor cell surface and the Yes-Associated-Protein (YAP) is tethered to the tumor cell membrane inducing dormancy. During trauma or inflammation, astrocytes can re-localize away from the dormant tumor cells and, in tumor cells, YAP can freely translocate into the nucleus and induce cell proliferation (Figure 1) [29].

In the bone marrow, the different types of HSC occupy at least two different niches, which can be hijacked by dormant tumor cells. The first is a PVN (see above) and the second is an endosteal niche composed of bone lining cells and osteoblast nestin-positive precursors such as mesenchymal stem cells, osteoblasts, osteoclasts, fibroblasts, and macrophages (Figure 1) [7,30,31]. These cells line the endosteal surface of the trabecular bone. Osteoblasts are responsible for the secretion of the main endosteal extracellular molecules, which are crucial for bone formation, such as osteopontin, collagen I, osteocalcin, and alkaline phosphatase. In bone marrow, the two niches have different roles in HSC. The PVN promotes HSC proliferation, differentiation, and mobilization, whereas the endosteal niche promotes HCS quiescence [31]. Different populations of osteoblasts are implicated in metastasis [32]. A population of osteoblasts makes heterotypic cadherin interactions with breast tumor cells inducing mTOR-dependent tumor cell proliferation and micrometastasis formation. A second population of osteoblasts is educated by breast tumor cells to express two ECM proteins, decorin (proteoglycan) and CCN3 (Nov), which are implicated in tumor cell proliferation inhibition in a mechanism dependent on p21 production [33]. In addition to osteopontin expression, these educated osteoblasts overexpress and secrete collagen I and MMP-3 (Figure 1) [33]. In contrast, osteoclast activation by breast tumor cells is implicated in bone ECM degradation and the release of different growth factors, such as TGF-β, which is implicated in tumor cell growth and metastasis development (Figure 1) [34,35]. Fibroblasts are quiescent cells in normal tissues. In the presence of cancer cells, they can be activated into a reversible cell phenotype called normal-activated fibroblasts [36]. Then, they acquire more properties and irreversibly become Cancer-Associated Fibroblasts (CAF). Fibroblasts and normally activated fibroblasts express factors and ECM proteins implicated in tumor cell dormancy, while most CAFs express factors and ECM proteins, such as tenascin C, which are generally implicated in tumor cell reactivation (Figure 1) [36].

The cell microenvironment of the different secondary organs targeted by the tumor cells is very specialized and can participate in the elaboration of specific niches. In bone marrow, Templeton and collaborators have postulated for an adipocyte niche. Nevertheless, it is not established if this niche can induce tumor cell dormancy [37].

The Alveolar Type 1 and 2 cells (AT1 and AT2 cells) play a key role in the lung tumor cell dormancy niche. In breast tumors, the interaction between the tumor cells and the AT1 cells activates the Secreted Frizzled-Related Protein 2 (SFRP2) protein, resulting in fibronectin fibril formation and tumor cell survival. Ectopic expression of SFRP2 in breast dormant D2-OR or 4T07 cells increases the lung metastatic burden and results in larger metastasis [38].

The liver niche is composed of hepatocytes and different non-parenchymal cells such as hepatic stellate cells, sinusoidal endothelial cells, or Kupffer cells. Exosomes are derived from the cells forming the liver niche and contain different miRNAs, miR-186 and miR-23a implicated in breast or prostate tumor cell dormancy. These miRNAs induce a mesenchymal to epithelial reversion of tumor cells [39]. Liver injuries activate hepatic stellate cells and their transformation into myofibroblasts. These later cells can secrete ECM molecules implicated in fibrosis and pro-inflammatory factors, such as Il-8, which increase the survival of breast tumor cells and promote tumor cell escape from dormancy [40]. Co-culture of sinusoidal endothelial cells with MCF-7 breast tumor cells increases the tumor cell proliferation, suggesting a possible role of these liver cells in the reactivation of the dormant tumor cells [41]. In a mouse model, an increase in Natural Killer (NK) cells has been observed in the liver niche, maintaining tumor cells dormant and preventing metastasis development [42]. During injuries, CXCL12 secretion by activated stellate cells pushes NK cells into quiescence and induces dormant tumor cell proliferation, contributing to dormant tumor cell reactivation and metastasis development [42].

The skeletal muscle is another secondary organ targeted by metastatic tumor cells and forms a permissive niche for cell dormancy. In this organ, few metastatic relapses were reported and they preferentially originate from lung, gastrointestinal, and breast primary tumors [43]. In this organ, most of the relapses appear after more than 5 years of total remission. For instance, a woman was reported to have a metastatic relapse in the skeletal muscle of breast cancer more than 20 years after remission [44]. These observations suggest that the skeletal muscle niche has a high antimetastatic potential. It was suggested that the vigorous oxidative stress in skeletal muscle could impair tumor cell proliferation [45]. However, alternative hypotheses such as the impact of ECM composition have been postulated, but this requires further investigations [43,46].

Additional organs are targeted by metastatic cells, such as cardiac muscle and spleen or thyroid tissue, suggesting the existence of a particular niche in each of these organs [43,46].

## 3. Models to Study Tumor Cell Dormancy

The mechanism of metastatic cell dormancy and reactivation was first studied in vivo with mice injected with tumor cells (orthotopically, subcutaneously, or in blood circulation). Metastatic organs were labeled for the proliferation marker Ki67 or for the growth arrest marker p27 [23]. Alternatively, the rate of proliferating cells was measured after injection of BrDu or EdU in mice [38]. Except for p27 labeling, this technique does not stain dormant cells: it only stains dividing cells. To circumvent this problem, tumor cells were labeled with an intracellular or membranous fluorescent vital dye before their injection. When cells proliferate, the mean fluorescence per cell decreases, while fluorescence intensity does not change in dormant cells [48].

To analyze dormancy and reactivation dynamics in tumor cells, an intravital lung imaging system called the Fluorescence Ubiquitination-based Cell Cycle Indicator (FUCCI) was used. With this system, cells in the G0/G1 phase are stained in red, while cells in the S and G2/M phases are stained in green [49]. In a different work, Ohta and collaborators subcutaneously xenografted mice with colorectal cancer organoids expressing the mVenus-p27^k-^ reporter gene to analyze in vivo dormancy. The fluorescent fusion protein was expressed only in the G0 and early G1 phases of the cell cycle [50]. Moreover, using three different reporter genes (mVenus-p27^k-^, hCdt1-iRFP, and hGeminin-TagBFP2), they were able to label cells in G0 (mVenus), G1 (RFP), S (BFP2), or G2/M (RFP and BFP2) phases of the cell cycle using different fluorescent colors, allowing the study of dormancy/reactivation kinetics [50]. Mouse models genetically modified to develop spontaneous tumors were also used to analyze tumor cell dormancy. As an example, the mouse MMTV-Her2-CFP model spontaneously develops cyan fluorescent-positive mammary tumors metastasizing in lungs and bone marrow, allowing the characterization of dormant cells [3].

Two alternative in vivo models can be used to study tumor cell dormancy. In the first, fluorescent tumor cells were injected intravenously into the vasculature of the chick chorioallantoidic membrane (CAM). After 2–3 days, cells extravazating in different organs could be visualized [51]. Fluorescent tumor cells can be injected in the sub-intestinal space of zebrafish embryos. The presence of extravazated dormant or proliferative cells can be analyzed over four days [23].

Organoids are also used to study tumor cell dormancy. Otha and collaborators used colorectal cancer organoids to characterize dormant tumor cells. Tumor dormancy was analyzed as previously described with mice models, ki67 labeling, the mVenus-p27^k-^ reporter gene, or the three different reporter genes (mVenus-p27^k-^, hCdt1-iRFP, and hGeminin-TagBFP2) (see above) [50].

In vivo models are highly relevant to study dormancy. Nevertheless, the presence of many interactions between cells or between cells and ECM makes the analysis of the role of a specific protein difficult. In vitro models bring the possibility to control and/or modify cells or ECM components to study dormancy or tumor cell reactivation. These in vitro assays are well described in recent reviews and books [52,53,54]. The simplest in vitro assay consists of the growth of tumor cells as a monolayer directly in a culture dish or in a culture dish coated with an ECM and then analyzing cell cycle division. Ghajar and collaborators cultivated endothelial cells and lung fibroblasts (or bone marrow mesenchymal stem cells) as monolayers to form the PVN, and after the addition of sparse tumor cells, they studied tumor cell dormancy [23].

In vitro tridimensional (3D) models have also been developed by growing tumor cells in biomaterial scaffolds such as collagen I, hyaluronic acid, fibrin, or matrigel. To recreate a specific niche, endothelial cells, fibroblasts, mesenchymal stem cells, and/or osteoblasts can be added to the system. In some assays, synthetic materials such as polyacrylamide, polyethylene glycol, polycaprolactone, and polyHEMA can substitute the biomaterials. Finally, some assays used a mix of natural and synthetic materials, allowing the control of physical constraints, such as stiffness, and the study of their effects on cell dormancy.

Moreover, a microfluidic system, commercially available under the name Liver-Chip (Emulate, USA), was used to create a liver niche and to study metastatic breast cancer cell dormancy [12]. This microfluidic system can be experimentally modified to incorporate synthetic materials such as polyethylene glycol [55], opening the way to investigations about the role of biological or synthetic materials in tumor cell dormancy. Finally, a human lung alveolar tissue was created in a microfluidic system and used as an orthotopic model for human non-small cell lung carcinoma development. With this model, Hassel and collaborators reported the presence of dormant tumor cells [56].

## 4. ECM as a Mediator of Cell Dormancy or Reactivation

**Figure 2:**
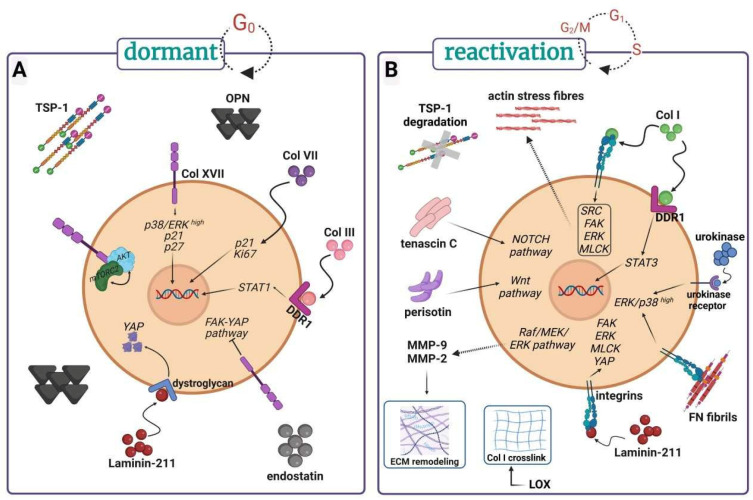
ECM components implicated in the maintenance of dormant tumor cells (**A**) or in the reactivation of dormant tumor cells (**B**). Figure created in BioRender. Redouté-Timonnier, C. (2024) https://BioRender.com/c31p125 (accessed on 1 December 2024).

In tumor dormancy, the ECM plays a critical role in regulating the balance between tumor cell quiescence and reactivation. Dormant cancer cells can remain inactive for long periods and are influenced by the mechanical properties, the composition, the stiffness, the remodeling, and the structure of ECM [57,58]. The role of the different components of ECM in tumor cell dormancy/reactivation is summarized in Figure 2.

### 4.1. Components of the ECM Involved in Cell Dormancy

#### 4.1.1. Collagens

Collagen protein family members are the most common components of ECM providing both structural strength and signaling to cells and tissues. Collagens types I (prominent member), II, III, V, XI, XXIV, and XXVII are fibril-forming collagens, while type VII, which does not belong to any class, can assemble and form anchoring fibril collagen [59,60]. Collagens XVII is part of the membrane collagen class, which also comprises types XIII, XXIII, and XXV [60], and collagen XVIII belongs to the multiplexin family, which is located within the basement membrane and can be cleaved into endostatin [59,60].

- Collagen III

Tumor-derived type III collagen is essential for inducing and maintaining cellular dormancy in vivo. This effect involves the binding of the Discoidin Domain Receptor family member 1 (DDR1) to type III collagen and the downstream activation of Signal Transducer and Activator of Transcription 1 (STAT1). Interestingly, the DDR1/STAT1 pathway promotes type III collagen expression to maintain dormancy [61]. This mechanism creates an ECM niche enriched in type III collagen, which promotes increased ECM curliness and remodeling, leading to cell dormancy sustained by DDR1. In addition, cells already in a dormant state are unaffected by external type III collagen produced by other cell types. Interestingly, tumor-dormant cells can stay in this state as long as they keep expressing collagen III [61]. Another study confirmed the role of type III collagen and demonstrated that a recombinant humanized type III collagen inhibits cellular autophagy, proliferation, and migration through DDR1 and promotes breast cancer cell dormancy [62]. However, recent data support the idea that autophagy is necessary for dormant tumor cells. It was shown that the inhibition of autophagy decreased the survival of breast cancer dormant cells [63]. Moreover, in the lungs, clusters of estrogen receptor α-positive (ER+) dormant breast tumor cells increase the expression of genes implicated in dormancy such as Col3A1, vimentin, and fibronectin [64].

- Collagen VII

In breast cancer, dormancy was induced in vitro by hypoxia. After investigating the role of different ECM molecules, the analysis revealed that collagen VII level was increased in the microenvironment of hypoxia-induced dormant cells compared to non-dormant cells maintained under normoxic conditions [65]. Breast cancer cells cultured as collagen VII-enriched 3D models presented a decreased proliferation rate associated with higher expression of cell cycle arrest marker p21 [65]. From 15 patient samples, a significantly higher amount of collagen VII expression was found in patients with low expression of the proliferation marker Ki67 [65].

- Collagen XVII

In colorectal cancer, upregulation of collagen XVII plays an important role in dormancy as it extends the quiescence status of these cells in a 3D model and enhances the resistance of colorectal cancer cells to chemotherapy. Through its intracellular domain, collagen XVII acts like a scaffold protein and allows crosstalk between the Target Of Rapamycin Complex 2 (mTORC2) and Protein Kinase B (AKT). This mechanism allows a transition from a proliferative to a dormant phenotype [66]. Upregulation of collagen XVII led to an increase in p21, p27, and the p38/ERK ratio, which are strong markers of a dormant phenotype [66,67,68]. In an organoid model, colon cancer stem cells expressing the Leucine Rich Repeat Containing G Protein-Coupled Receptor 5 (LGR5) and p27 are in dormancy and this state is regulated by cell–matrix interactions. In colon cancer stem cells, collagen XVII inhibits the Focal Adhesion Kinase (FAK)–YAP pathways and blocks p27-positive cells from re-entering into the cell cycle [50]. A treatment, such as chemotherapy, induces ECM remodeling. Collagen XVII proteolysis can trigger the dormant LGR5 and p27 positive cancer stem cells to re-enter the cell cycle and, consequently, tumor regrowth [50]. Interestingly, collagen XVII was recently described as a positive marker for dormant colon cancer stem cells [50].

- Collagen XVIII-Endostatin

The secretion of MMPs by different types of cells, such as macrophages, can lead to the release of anti-angiogenic factors, including endostatin, into the ECM, which can affect the dormancy state of tumor cells [69,70]. O’Reilly and colleagues showed that administration of endostatin inhibits the growth of lung primary tumors and metastasis in mice, suggesting that endostatin therapy strongly supports tumor dormancy [71]. In addition, when tumor cells are in an anti-angiogenic dormancy state, perivascular tumor cell expansion is blocked as a result of the balance between pro- and anti-angiogenic factors such as endostatin [69]. Endostatin inhibits angiogenesis at several levels in vivo, including perivascular cell recruitment [69,72], and can lead to inhibition of endothelial cell migration in vitro and tumor growth in vivo [72].

#### 4.1.2. Glycoproteins

- Fibronectins

Fibronectin fibrils can be considered as a pro-dormancy or as a reactivated component of ECM [73,74]. As a pro-dormancy factor, Barney et al. showed that breast tumor cells entering in dormancy consistently produce and assemble fibrillar fibronectin matrix via integrin αvβ3 and α5β1 adhesion, ROCK-generated tension, and TGF-β stimulation [73]. In this in vitro assay, ubiquitous p-ERK and heterogeneous p-p38 are expressed by tumor cells, suggesting a balance between cells in proliferation and quiescent cells. This observation between the two types of cells shows that it is more a tumor dormancy than a tumor cell dormancy [73,75].

- Laminins

As shown before, laminin-211, deposited by astrocytes, induces quiescence in DTCs in the brain through a mechanism dependent on the cytoplasmic sequestration of YAP by dystroglycan [29]. However, proteolytic fragments of some laminins were implicated in tumor cell reactivation and exit from dormancy (see Laminins in Section 4.2.2).

- Thrombospondin-1

As mentioned earlier, a stable microvasculature forms a dormant niche and maintains prolonged tumor cell dormancy through TSP-1 [23]. At sprouting endothelial tips, the tumor-suppressive properties of the microvascular endothelium are lost, and a decrease in TSP-1 and an increase in pro-tumoral ECM molecules, as periostin, are observed [23]. These findings align with the work of Catena et al., who discovered that in the lung pre-metastatic niche, non-metastatic tumors stimulate the expression of the TSP-1 in the recruited pro-metastatic bone marrow cells to transform these cells into metastasis-inhibitory cells. This impaired the outgrowth of metastatic tumor cells [76]. In human melanoma xenograft mice exposed to radiation treatment, the re-growth of dormant micro-metastases could be prevented by treating them with exogenous TSP-1 [77]. Moreover, primary tumors suppress the growth of their spontaneous pulmonary micrometastases by secreting TSP-1 into the blood, suggesting that TSP-1 could leave the cells in a dormant state [77,78].

TSP-1 binds to several receptors, such as CD47 or different integrins β1. The receptor implicated in tumor cell dormancy is not yet characterized [79].

- Osteopontin

To support tumor cell dormancy in bone marrow, osteopontin secreted by osteoblasts anchors leukemic blasts within the endosteal niche in an integrin α4β1-dependent mechanism. In turn, acute lymphoblastic leukemia cells specifically secrete osteopontin when localized into the endosteal niche in vivo. This interaction induces the cell cycle exit of leukemic blasts, protecting them from cytotoxic chemotherapy. These data suggest that osteopontin can expand the quiescent niche and reinforce the dormant phenotype [80].

#### 4.1.3. ECM Stiffness

Matrix stiffness has been reported to play a significant role in inducing dormancy in tumor-repopulating cells (TRCs). Specifically, stiff fibrin gels (1050 Pa)—indicating increased 3D matrix stiffness—can drive TRCs from melanoma cells into a dormant state. By measuring the level of the Ki67 marker, complementary data revealed that exposure of melanoma cells to a stiff fibrin matrix activates the cell division cycle 42 (CDC42)/TET methylcytosine dioxygenase 2 (TET2) pathway and induces cell dormancy [81]. A recent study showed that in adhesive conditions, increasing the crosslink density of hydrogels induces the formation of cluster cells. A strong increase in hydrogel crosslink density leads to a balance between death and proliferation, which is similar to micrometastasis dormancy. However, when matrix adhesiveness is absent, cancer cells enter tumor cell dormancy regardless of the crosslink density [82]. Both studies show that stiffness is not the only physical constraint for tumor cell dormancy. Indeed, other constraints such as adhesion are also important.

Immune cells such as tumor-associated macrophages (TAMs) can influence ECM stiffness [83]. Interestingly, Transglutaminase 2 (TGM2), which is responsible for fibronectin and Col I crosslinking, thereby facilitating ECM stiffness, is highly expressed by TAMs [84]. This suggests the role of TAMs in tumor cell dormancy.

### 4.2. ECM Components Involved in the Reactivation of Dormant Tumor Cells

#### 4.2.1. Collagens

- Collagen I

Fibrosis, a high level of collagen I in the metastatic environment, induces the transition from a dormant to a proliferative state of mammary cancer cells [85,86]. This switch can be induced through integrin β1 and activation of SRC, FAK, ERK, and Myosin Like Chain Kinase (MLCK) and lead to the phosphorylation of MLC and actin stress fiber formation [85,87]. In addition, mammary cancer metastatic lesions are associated with significant depositions of fibronectin and collagen I. These mammary cancer cells, cultured in a 3D system supplemented with fibronectin and/or collagen I, undergo a quiescence-to-growth state transition. Interestingly, secreted mediators generated by CD11b^low^ macrophages prevent dormant mammary cancer cells from escaping dormancy. These mediators inhibit fibroblast differentiation to myofibroblasts and collagen I expression [88]. Together, these studies illustrate that collagen I production as well as its interaction with integrin β1 is crucial for the switch from dormancy to metastatic outgrowth in the lungs [85,87,88].

In breast cancer dormant cells, the interaction of DDR1 with collagen I regulates self-renewal and metastatic reactivation via Signal Transducer and Activator of Transcription 3 (STAT3) activation. Mechanistically, Transmembrane 4 L6 Family Member 1 (TM4SF1) mediates the non-canonical DDR1 signaling by linking DDR1 to Protein Kinase C-alpha (PKCα), which activates Janus Kinase 2 (JAK2), resulting in STAT3 phosphorylation and activation. This non-canonical signaling cascade induces the expression of SRY-Box Transcription Factor 2 (SOX2) and NANOG, promotes the maintenance of cancer stem cell properties and drives metastatic reactivation in the lung, bone, and brain. In addition, immunohistochemical analysis revealed that the cancer cells within metastatic sites exhibit significantly higher levels of nuclear phospho-STAT3 compared to those in primary tumors [89].

In breast cancer, the LOX-mediated collagen I crosslink increases tumor cell proliferation and enhances metastatic colonization and growth [90]. Furthermore, LOX increases matrix stiffness through its collagen crosslinking activity, which directly influences cellular behavior and leads to tumor cell proliferation [90]. Moreover, LOX released by hypoxic tumor cells at the primary site can induce collagen crosslinking at distant pre-metastatic niches, thereby contributing to the establishment and maintenance of these niches [91].

#### 4.2.2. Glycoproteins

- Fibronectin

In some conditions, fibronectin can reactivate dormant tumor cells. In human head and neck carcinoma, breast cancer, prostate cancer, melanoma, and fibrosarcoma cell lines, the level of phospho-ERK and the ERK/p38 activity ratio can predict the proliferation or the dormancy of cells. A high ERK/p38 activity ratio favors tumor growth, whereas a low ERK/p38 activity ratio induces dormancy [67]. A high ERK/p38 activity ratio is induced by high urokinase receptor expression, which leads to the activation of integrin α5β1 and epidermal growth factor receptor. The ERK/p38 ratio is enhanced by urokinase binding to the urokinase receptor and fibronectin binding to integrin α5β1. Moreover, integrin activation can organize fibronectin into insoluble fibrils, suppressing p38 activity and tipping the balance in favor of ERK activity and tumor growth [74].

- Laminins

In breast cancers, lung inflammation activates Neutrophil Extracellular Traps (NETs). The NET-associated proteases, neutrophil elastase and MMP-9, remodel the ECM by cleaving laminins, particularly laminin-111, -211, -411, and -511. A new laminin epitope, revealed by the protease cleavage, binds and activates integrin α3β1, which triggers FAK/ERK/MLCK/YAP signaling in cancer cells, leading to the reawakening of dormant breast cancer cells in the lung [49]. During NET, TSP-1 regulates the effect of cleaved laminin-111 and decreases tumor cell reactivation [49].

In breast cancer, MMP-9 can also be increased by hyperactivation of the Rapidly Accelerated Fibrosarcoma (RAF)/MEK/ERK pathway. In turn, MMP-9 induces laminin-111 proteolysis and tissue architecture perturbation. The loss of laminin-111 integrity leads to malignant progression and tissue polarity disruption [92].

- Periostin

In a genetic mouse model of breast cancer, TGF-β3 increases periostin expression and secretion in lung DTCs, which in turn activates Wnt signaling to allow cancer stem cell maintenance. In periostin-inactivated mice, metastasis colonization is inhibited [93]. Moreover, as previously described above, at sprouting endothelial tips, the tumor-suppressive properties of the microvascular endothelium are lost and an increase in periostin is observed that can promote breast cancer outgrowth [23].

In human colon cancer, periostin can activate the AKT/PKB signaling pathway through the integrins α_v_β_3_ and increase both tumor and endothelial cell survival, which promotes tumor metastasis of colon cancer. This provides a molecular explanation for periostin pro-survival role in tumor progression by facilitating angiogenesis and the formation of metastatic colonies. Both integrins α_v_β_3_ and the AKT/PKB pathway are key contributors to cell survival and tumorigenesis [94].

- Tenascin C

As described above, the disruption of vascular homeostasis allows the induction of neovascular sprouting in the PVN and perturbs endothelial architecture. This leads to a deposition of ECM molecules such as tenascin C that promotes micrometastatic outgrowth [23]. Tenascin C was shown to promote breast cancer cell infiltration into the lung and to support the metastasis-initiating ability of these tumor cells. To do this, tenascin C enhances the expression of LGR5, a Wnt target, and of Musashi RNA binding protein 1 (MSI1), which improves the performance of the Neurogenic Locus Notch Homolog Protein 1 (NOTCH1) pathway [95]. Transgenic mice overexpressing c-myc in the mammary gland develop slow-rate lung micrometastasis. In contrast, transgenic mice overexpressing c-myc and the angiogenic factor VEGF in the mammary gland result in high rates of pulmonary macrometastasis. Comparing the two transgenic mice revealed that VEGF induces changes in tissue architecture and gene expression. A gene signature associated with human breast cancer lung metastasis was identified and included key markers such as tenascin C and MMP-2. Downregulation of tenascin C significantly impaired the ability of cancer cells to disseminate and colonize the lungs and reduced tumor relapse [96].

## 5. ECM Remodeling Factors

- Metalloproteinases (MMPs)

Different MMPs are implicated in tumor development and some of them, essentially the gelatinase MMP-2 and MMP-9, have a role in dormant cell reactivation. Barney et al. showed that cells that can proliferate after dormancy required the ability of MMP-2 and -9 to degrade the fibronectin matrix [73]. Pro-MMP-2 overexpression enhances the metastatic potential of breast cancer in vivo and significantly increases cell invasiveness in vitro. This overexpression is associated with a higher incidence of breast cancer metastasis to organs such as the bone, brain, liver, and kidney, as well as an increase in the metastatic burden in the lungs. These findings highlight the critical role of MMP-2 in promoting cancer progression and metastasis across multiple tissues [97]. Expressed in lung fibroblasts, MMP-2 has a role in breast cancer progression and enhances tumor cell proliferation in lung metastasis. Indeed, active fibroblasts can release proteases and cytokines that induce the production of TGF-β1, which is an important mediator of the transition from a quiescent to a reactive stroma [98].

- Lysyl oxidase and Lysyl oxidase-like

The Lysyl Oxidase (LOX) and Lysyl Oxidase-Like (LOXL) family contains various members, LOX and LOXL1–4, characterized by a catalytic activity implicated in remodeling and cross-linking the components of the ECM [99]. LOX-mediated collagen I crosslinking can change cell behavior and be associated with tumor cell dormancy exit (see above) [90]. LOX expression can be modulated by hypoxia. In triple-negative breast cancer (TNBC), LOX was identified as an ECM remodeler as a result of hypoxia. Inhibiting LOX was shown to decrease collagen crosslinking and fibronectin assembly, thereby reducing ECM stiffness [100]. Moreover, paclitaxel-based chemotherapy induces the expression of LOX by CD8+ T cells [101]. LOX can, also, be upregulated by cytokines such as Tumor Necrosis Factor-alpha (TNF-α) secreted by CD8+ T cells [102]. LOXL1 expression was strongly associated with Interleukine-4 (IL-4), another T lymphocyte cytokine. The association between LOX and cytokines secreted by CD8+ T cells, such as TNF-α or IL-4, reinforces the fact that immune cells have a strong impact on ECM remodeling and tumor cell dormancy exit.

## 6. ECM Is Implicated in Autophagy and Immune Evasion in Tumor Cell Dormancy or Reactivation

### 6.1. ECM and Autophagy

Tumor cell dormancy can activate various survival mechanisms such as autophagy in response to environmental stresses. As an example, the dormant breast cancer cell line D2.0 R was reported to use autophagy in vitro and in vivo to survive [63]. Interestingly, dormant breast cancer cells cultured in a basal membrane extract displayed an increased expression of autophagy markers such as microtubule-associated protein 1 A/1B-light chain 3 (MAP1LC3, known as LC3) and Lysosomal-Associated Membrane Protein 1 (LAMP1) [103,104]. As previously described, collagen I can trigger the regrowth of dormant cells [85,86,87,88]. When the dormant breast cancer cells D2.OR were grown in basal membrane extract enriched with collagen I, the levels of LC3 and LAMP1 were significantly reduced [63]. The authors also showed that a particular type of autophagy, known as mitophagy, can be used by dormant breast cancer cells to survive under these conditions. Mitophagy is described as the degradation or engulfment of damaged or excess mitochondria [63]. One major pathway involved in this mechanism is the PINK1 pathway, which has been activated in dormant breast cancer cells. Thus, clearing damaged mitochondria seems to be a key mechanism in the survival of dormant breast cancer cells [63].

A loss of integrin β1 mediated-cell adhesion leads to cell detachment from ECM and autophagy. In cells, the loss of ECM adhesion induces stress that activates the Protein kinase-like ER kinase (PERK) pathway. This leads to the activation of AMP-activated protein Kinase (AMPK), which mediates the induction of autophagy. Both PERK and AMPK activation results in the suppression of mTOR signaling [105,106]. PERK is essential for activating the Liver kinase B1 (LKB1)/AMPK complex, leading to tuberous sclerosis complex 2 (TSC2)-mediated mTOR inhibition. Interestingly, the authors provide evidence of a “fast kinetic signaling” from PERK and mTOR, which can rapidly provide an Adenosine TriPhosphate (ATP) source for the cells [105,106]. Since the ECM undergoes significant changes during cancer progression, dormant cells could use ECM-detachment-induced autophagy as a pro-survival strategy. Indeed, as ECM detachment is associated with a stressful environment and the ECM is continuously modified during cancer progression, this mechanism could allow cells to enter dormancy and survive through autophagy [107].

### 6.2. ECM and Immune System

Dormant cells can use immune evasion strategies to persist and escape immune surveillance. This immune evasion may be facilitated by ECM remodeling. In an in silico study, dormant TNBCs were found to preferentially express ECM pathways as “ECM proteoglycans”, “laminin interactions”, “integrin cell surface interactions”, or “collagen formation”. This dormant-associated ECM signature was also linked to an immunosuppressive TME enriched in CAFs, endothelial cells, and TAMs [108].

In inflammatory conditions, particularly within pulmonary inflammatory diseases, CXC chemokines such as IL-8 have been identified as key attractants for neutrophils. Interestingly, collagen-derived peptides, such as the proline-glycine-proline (PGP) tripeptide, mimic CXC chemokines interacting with C-X-C motif chemokine receptor 1 (CXCR1) and C-X-C motif chemokine receptor 2 (CXCR2), thus directing neutrophils to regions of ECM remodeling. Notably, exposure to lipopolysaccharides (LPS) can cause the liberation of PGP in vivo [109]. This mechanism might also be relevant during cancer progression when inflammation is involved [110]. Given that neutrophils are known to release matrix remodeling enzymes such as elastase or MMPs, their presence could contribute to ECM remodeling, leading to the release of ECM components [111]. These findings suggest the critical role of ECM in inflammation, promoting the reactivation of dormant tumor cells.

## 7. Treatment Strategies

Three main different approaches to target cancer cell dormancy have been considered. In the first, cancer cells will be maintained in dormancy. In the second, dormant tumor cells will be reactivated to increase their sensitivity to anti-proliferative drugs. In the third, dormant tumor cells will be targeted by specific drugs or inhibitors. Besides these approaches, other strategies can be considered. In this section, we will focus more on strategies in which ECM components are implicated.

- Maintenance of tumor cell dormancy

Various therapies have been proposed to force and maintain tumor cell dormancy. Most of them try to inhibit intracellular pathways implicated in cellular proliferation (i.e., ERK signaling) or to activate cellular signaling implicated in cellular dormancy (i.e., p38 signaling) [74]. Another possibility is to inhibit the cell cycle by using CDK antagonists. In breast HR+ (Hormone Receptor) HER2- (non-amplified) cancers, CDK4/6 inhibitors were used in clinical trials as first- or second-line therapy or as adjuvant therapy with different efficacy [112]. Used in first- or second-line therapy and in combination with hormonal therapy, palbociclib, abemaciclib, or ribociclib treatments significantly increase patient-free survival (PFS) [112]. As adjuvant therapy and, also, in combination with hormonal therapy, CDK4/6 inhibitors gave contrasting results. A two-year palbociclib treatment on patients with stage II-III disease did not affect the 4-year invasive disease-free survival (iDFS) of patients but had increased toxicity. In contrast, ribociclib treatment has a beneficial effect [112]. In these breast cancer clinical trials, regimens with CDK4/6 inhibitors are short (only one or two years), but satisfactory benefits were obtained for patients.

To maintain tumor cell dormancy, it is tempting to modulate the expression of ECM molecules implicated in tumor cell dormancy or reactivation. Two kinds of drugs or chemical compounds can be used: the first increases expression of ECM molecules (i.e., collagen III, endostatin…) keeping tumor cell dormancy, and the second decreases ECM molecules, inducing tumor cell reactivation (i.e., collagen I, periostin, tenascin c…). Fibrosis is characterized by a huge increase in collagen I expression, which induces the reawaking of dormant cells [85]. Lung fibrosis can be targeted by the tyrosine kinase inhibitor nintedanib and recently, Reinecke and collaborators showed that this drug inhibits osteosarcoma lung metastasis [113]. It will be interesting to know if nintedanib can maintain the dormancy of tumor cells, which preferentially metastasize to the lung, such as in kidney cancers.

Integrin β1 is a subunit of different integrins, which bind many ECM proteins implicated in the reactivation of dormant tumor cells. The inhibition of integrin β1 expression by shRNA strategy was shown to maintain breast tumor cells dormant as it was experimentally shown in lungs [85]. Several inhibitors of integrin β1 such as ATN-161, volociximab, and JSM6427 may be used in cancer therapy [114]. With these inhibitors, several clinical trials against metastatic melanoma or renal cell carcinoma are ongoing, but the results are not yet published.

The parathyroid hormone (teriparatide, PTH (1–34)) is used in osteoporosis to increase osteoblast differentiation and bone mass. Intermittent injections of PTH preserve bone architecture after breast tumor cell grafting and the bone metastasis number is dramatically reduced [115]. Osteoblasts are the main cells expressing osteopontin, and this ECM protein is implicated in leukemic cell dormancy in the endosteal niche. It is tempting to speculate that PTH could be used to increase osteoblast differentiation to maintain the ECM endosteal niche and to keep leukemic tumor cells dormant [80].

Treatments to maintain tumor cell dormancy in their niche can stop metastasis development and prevent recurrences. Nevertheless, the treatment should be kept uninterrupted during the entire life of the patient, and it is likely that dormant cells will acquire mutations implicated in cell proliferation reactivation with time.

- Reactivation of dormant tumor cells

Classical cancer treatments used anti-proliferative drugs, which are inefficient against dormant tumor cells. In contrast, reactivated dormant tumor cells can become sensitive to anti-proliferative drugs. In nasopharyngeal carcinoma, radiation or chemotherapy induces the formation of dormant polyploid giant cancer cells by a mechanism of autophagy. In a mouse orthotopic model, autophagy inhibition induced by hydroxychloroquine followed by cisplatin treatment abolished the formation of metastasis and improved mouse survival [116]. A clinical trial using hydroxychloroquine before radiotherapy or chemotherapy in advanced nasopharyngeal carcinoma patients is currently under investigation (NCT06389201).

Another approach to reactivate dormant tumor cells is to modify the metastatic niche. Dormant myeloma cells established in the endosteal niche are protected against melphalan, an alkylating drug used in myeloma treatment. Osteoclast stimulation activates dormant myeloma cells, which leads to metastasis growth. Unfortunately, the authors did not treat mice with melphalan to kill the proliferated cells [35]. Direct manipulation of the ECM of the niche can be used to reactivate tumor cells. In a model of leukemic cell engraftment in mice, anti-osteopontin antibodies can reactivate dormant leukemic cells. Cytosine arabinoside (Ara-C), commonly used in leukemia treatment, synergizes with anti-osteopontin antibodies to decrease the number of dormant cells in the endosteal niche without macrometastasis development [80]. The PVN may be targeted with ECM antibodies to increase angiogenesis. Disruption of vascular homeostasis allows the induction of neo-vascular sprouting and perturbs endothelial architecture, leading to dormant cell reactivation [117].

This strategy has the advantage of killing the dormant tumor cells with a combination of at least two drugs, one for the reactivation of dormant cells and the second to eliminate proliferative cells. However, all the dormant cells are not reactivated, resulting in the presence of a residual pool of dormant cells. Moreover, the reactive cells could be more aggressive and/or resistant to the anti-proliferative drug, resulting in worse metastasis development.

- Targeting dormant tumor cells

The last strategy is to kill dormant tumor cells. In breast cancer cells, Src Family Kinase (SFK) inhibition prevents dormant cell activation from external stimuli in the lungs and decreases metastasis burden but does not eliminate the dormant tumor cells. In contrast, SFK and MEK1/2 inhibitions induce the apoptosis of dormant cells and decrease lung metastasis [118].

In an in vitro PVN model, blocking the interaction between breast tumor cells and ECM with antibodies against integrins β1 and/or αvβ3 sensitizes cells to doxorubicin. In different in vitro and mouse models, integrins β1 or/and αvβ3, partially inactivated with shRNA in tumor cells or antibodies, sensitize dormant tumor cells to doxorubicin or to doxorubicin plus cyclophosphamide. Consequently, the metastasis burden was inhibited if mice were treated with anti-β1 or/and αvβ3 antibodies and treated with doxorubicin plus cyclophosphamide [27].

This strategy is very promising if 100% effective. However, if some dormant cells are not killed, residual cells can become very aggressive after reactivation.

- Combination treatment

A phase II clinical trial called PALAVY using different combinations comprising the inhibitor of autophagy hydroxychloroquine, the CDK4/6 inhibitor palbociclib, and the immune checkpoint inhibitor PD-L1 avelumab is currently under investigation in early ER+ breast cancer patients (NCT04841148). The different drugs will be applied after definitive treatment and aim to reduce the incidence of recurrent metastasis in ER+ breast cancers by targeting both dormant and reactivated dormant cells.

ECM molecules or their receptors on tumor cells are promising targets to eliminate dormant cancer cells. Nevertheless, more research is needed on different cancer models. A treatment could be promising for one cancer, but not for another one. In breast cancer, integrin β1 inhibition keeps cells in a dormant state [49], but the same treatment in leukemia reactivates dormant cells [80], certainly because the cells grow in different niches.

## 8. Conclusions—Perspectives

For most cancers, but not all, metastasis is the major cause of death. Conventional and new therapies have considerably increased the disease-free survival and relapse-free survival of patients. However, the presence of dormant cells in different niches in bone marrow and in different organs has emphasized the formation of late or very late metastasis. So far, no specific markers of dormant cells have been characterized. This is a key task in the study of tumor cell dormancy in cancer and the characterization of metastatic niches. Single-cell transcriptomics and spatial omics are new powerful tools that could help research identify such dormant tumor cell markers. However, as for cancer stem cells, it is likely that dormant markers are different from one metastatic niche to another, depending on the cancer cell type and organ affected by the primary tumor.

Depending on the tumor, dormant cells are found in a particular niche, showing direct or indirect interactions between the niche and the tumor cells. The different ECM components of the metastatic niche are important molecules implicated in the induction of tumor cell dormancy as well as in the reactivation of dormant cells. During the last decade, the research community has gathered important findings in breast cancer about ECM composition in the endosteal and peri-vascular metastatic niches. However, it is unknown if ECM composition in these niches is different or similar in all cancers. Moreover, for one primary tumor type, it is unknown if the ECM composition in one particular niche will be the same in all metastatic secondary organs. For one cancer type, is PVN identical in all metastatic secondary organs? Most cancers can metastasize in the bone marrow where at least two closely related niches, the PVN and the endosteal niche, can be found. The composition of these two niches is different and the interactions between each niche and the tumor cells are particular. As an example, integrin β1 seems to have an antagonistic role whether the tumor cells are in the endosteal niche or in the PVN.

Until now, ECM receptor research mainly focused on integrins. However, additional ECM receptors exist, such as DDRs, and likely play a role in dormancy or in tumor cell reactivation. Deciphering the role of these ECM receptors might facilitate the characterization of intracellular signaling implicated in these mechanisms and might lead to more curative therapeutic strategies aiming to induce or reawake dormant cells and lessen the rate of patients relapsing.

Exploring the different niches with high anti-metastatic potential, such as cardiac or skeletal muscles, is of great interest to understand why few metastases develop in these organs. As postulated by Crist and Ghajar, the ECM composition is, certainly, implicated in this phenomenon [46].

A better knowledge of the role of each ECM component on tumor dormancy and reactivation could bring new promising alternatives to kill or keep quiescent dormant tumor cells and, consequently, eradicate late metastasis and decrease the relapse rate.

## Data Availability

Not applicable.
